# A Small Range Six-Axis Accelerometer Designed with High Sensitivity DCB Elastic Element

**DOI:** 10.3390/s16091552

**Published:** 2016-09-21

**Authors:** Zhibo Sun, Jinhao Liu, Chunzhan Yu, Yili Zheng

**Affiliations:** 1 School of Technology, Beijing Forestry University, Beijing 100083, China; szbandlzl@bjfu.edu.cn (Z.S.); ycz_vicky@bjfu.edu.cn (C.Y.); zhengyili@bjfu.edu.cn (Y.Z.); 2 Engineering Training Center, Beihang University, Beijing 102206, China

**Keywords:** six-axis accelerometer, parallel mechanism, double cantilever beam, sensitivity analysis

## Abstract

This paper describes a small range six-axis accelerometer (the measurement range of the sensor is ±g) with high sensitivity DCB (Double Cantilever Beam) elastic element. This sensor is developed based on a parallel mechanism because of the reliability. The accuracy of sensors is affected by its sensitivity characteristics. To improve the sensitivity, a DCB structure is applied as the elastic element. Through dynamic analysis, the dynamic model of the accelerometer is established using the Lagrange equation, and the mass matrix and stiffness matrix are obtained by a partial derivative calculation and a conservative congruence transformation, respectively. By simplifying the structure of the accelerometer, a model of the free vibration is achieved, and the parameters of the sensor are designed based on the model. Through stiffness analysis of the DCB structure, the deflection curve of the beam is calculated. Compared with the result obtained using a finite element analysis simulation in ANSYS Workbench, the coincidence rate of the maximum deflection is 89.0% along the *x*-axis, 88.3% along the *y*-axis and 87.5% along the *z*-axis. Through strain analysis of the DCB elastic element, the sensitivity of the beam is obtained. According to the experimental result, the accuracy of the theoretical analysis is found to be 90.4% along the *x*-axis, 74.9% along the *y*-axis and 78.9% along the *z*-axis. The measurement errors of linear accelerations *a_x_*, *a_y_* and *a_z_* in the experiments are 2.6%, 0.6% and 1.31%, respectively. The experiments prove that accelerometer with DCB elastic element performs great sensitive and precision characteristics.

## 1. Introduction

Due to significant advantages in terms of stiffness, payload and precision, a parallel mechanism is generally applied in the design of six-axis accelerometers [1,2,3,4]. Six-axis accelerometers have been widely used for applications such as head impact sensors [5] and structural health monitoring [6]. Four identical T-shaped bars were applied to a novel six-component force sensor by Liu Sheng et al. [7]. The Gough–Stewart platform is a typical parallel mechanism in the field of sensors [8,9,10,11,12], and this platform was adopted as a six-dimensional force/torque sensor by Kerr in early research [13]. To improve the system, Dwarakanath et al. developed a ring-type sensing element for the Stewart platform [14,15]. Seibold et al. designed a six-dimensional force sensor with elastic joints for minimally invasive surgery [16], and Nicholas developed a sensor for biomechanical measurement [17]. Gao Feng designed a micro six-dimensional force sensor applied to the finger and wrist of a robot [18], while Ranganath et al. designed a directionally sensitive force/torque sensor, in a near-singular configuration [19]. A ring-type sensing element was applied to the system, and the joints connecting the legs to the base and platform were replaced by flexural hinges. Gao Zhen et al. designed a novel architecture of a 3RRPRR(a parallel mechanism with revolute and prismatic pairs) fully decoupling parallel mechanism to replace the Gough-Stewart platform, which showed good results in terms of isotropy, stiffness and sensitivity [20]. With the development of the microelectronic technology, Microelectromechanical systems (MEMS) are developed for convenience [21,22], such as ADI ADXL345 accelerometer and ST TA0343 three-axis MEMS. Advantages of MEMS are small size, high sensitivity and accuracy in measurement. However, the development and advancement of MEMS devices require a substantial design and fabrication infrastructure. The objective of this work is to develop a silicon-independent accelerometer that can be manufactured with a substantially less complex infrastructure. Compared with development of a MEMS, that of a mechanical accelerometer performs better in reliability, simple construction and economy.

In the measurement process, real-time detection and observation depends on dynamic characteristics. To obtain better dynamic characteristics, a number of studies have focused on the structure of the accelerometer. Based on Kane’s method, Yun Yuan and Yao Jiantao established a mathematical model of a six-axis accelerometer with a parallel mechanism [23,24]. Detailed research by You Jingjing et al. yielded a dynamic decoupling model of the system using a Hamiltonian dynamics equation [25].

Double cantilever beam (DCB) structure is a normal elastic-sensitive element. A. B. deMorais applied three-dimensional finite element analyses to the development of a metal adhesively-bonded double cantilever beam (DCB) and analyzed the critical strain energy release rate of the DCB [26,27]. DCB structures are also used in sensor applications. Wang Jianhua applied a DCB structure to a strain-measurement sensor and analyzed the strain of the DCB [28]. Yu Weijing et al. designed an elastic sensitive element with a DCB structure [29]. Largely nonlinear dynamical traits of cantilever beams with local nonlinearities and double cantilever beams have been shown in the literature before [30,31]. These nonlinear effects likely provide an advantage in increasing their sensitivities.

Because of the good 6-DOF (degree of freedom) characteristic of steward platform and the good elastic characteristic of the DCB structure, this paper comprehensively investigates a novel six-axis accelerometer combined with the steward platform and DCB structure. At the first, the paper introduces the structure of the small range six-axis accelerometer. DCB-sensing elements are applied to improve the sensitivity of the system. Secondly, the mass matrix and stiffness matrix are obtained based on a partial derivative calculation and a conservative congruence transformation (CCT). The dynamic model is established based on the Lagrange equation. Then, by simplifying the structure of the system, a model of the undamped free vibration is obtained. Finally, through stiffness and strain analysis of the DCB structure, theoretical analysis of the deformation and sensitivity of the beam are undertaken. Finite element analysis (FEA) simulations and experimental results from a prototype accelerometer are applied to prove the validity and reliability of the research methods.

## 2. Structure of the Accelerometer

**Figure 1:**
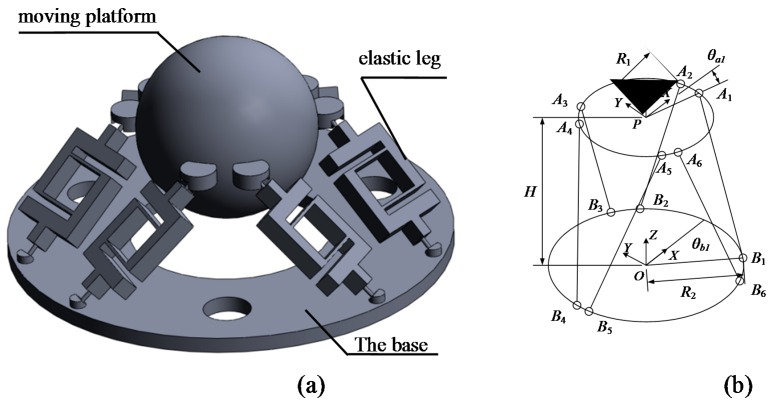
Structure of the six-axis accelerometer: (**a**) three-dimensional model of a small range six-axis accelerometer; and (**b**) theoretical model of the six-axis accelerometer.

As shown in Figure 1a, the preliminary design of the accelerometer consists of three parts: A moving platform, elastic legs and a base. The moving platform is a spherical structure serving as the inertial mass of the system, and the base is fixed to the measured object. Six elastic legs are connected to the moving platform and the base with flexural hinges. The moving platform has six active degrees-of-freedom (DOF) caused by the deformation of the elastic legs. If an external acceleration is applied to the platform, the axial forces and deformation of the legs required to keep the moving platform in equilibrium are obtained.

Figure 1b shows the theoretical model of the accelerometer. The geometrical center of the fixed base is located at the points ***O*** where the base coordinate *O*-*XYZ* is established. The base connection points are ***B**_i_*, *i* = 1, 2, …, 6, and *R_B_* denotes the radius of the concentric circles on the base. *θ_bi_* denotes the directional angles of connection points with respect to the *X* axis of the coordinate *O*-*XYZ.* A point ***P*** is located at the center of the moving platform. Local coordinates *P*-*XYZ* of the moving platform are established at point ***P*** and the radius of the moving platform is *R_A_*. The connection points of the moving platform are ***A**_i_*, *i* = 1, 2, …, 6. On the moving platform, *θ_ai_* denotes the directional angles of the connection points with respect to the *X*-axis of the coordinate *P*-*XYZ*. *H* denotes the distance between the moving platform and the base.

## 3. Dynamic Analysis

### 3.1. Lagrange Equation for the System

For a general parallel manipulator, the number of associated generalized coordinates is usually equal to the DOF of the moving platform [32]. In this paper, the generalized coordinates in the general model are set to $q={\left[\begin{array}{cccccc} q_{x} & q_{y} & q_{z} & q_{\alpha } & q_{\beta } & q_{\gamma } \end{array}\right]}^{T}$, which represent the displacements and rotations of the moving platform along *X*-, *Y*- and *Z*-axes. 

The system-generalized speed $\dot{q}={\left[\begin{array}{cccccc} {\dot{q}}_{x} & {\dot{q}}_{y} & {\dot{q}}_{z} & q_{\alpha } & q_{\beta } & q_{\gamma } \end{array}\right]}^{T}$ is defined as the time change rate of the generalized coordinates in the base coordinate system.

The local coordinate of the moving platform with respect to the base is described by a translation vector ***P*** and rotation matrix ***R***, and the initial coordinate of the vector ***P*** is [0,0,H]^T^. In the base coordinate *O*-*XYZ*, the position and pose matrix of the moving platform is written as:

(1) $$S=\left[\begin{array}{cc} diag\left(P\right) & R \end{array}\right]$$

In Cartesian coordinate system, *P*-*XYZ*, vectors of points ***A**_i_* are written as:

(2) $$A_{i}={\left[\begin{array}{ccc} R_{A}\cdot \cos\;\theta_{ai} & R_{A}\cdot \sin\;\theta_{ai} & 0 \end{array}\right]}^{T}$$

In the Cartesian coordinate system, *O*-*XYZ*, vectors of points ***B**_i_* can be written:

(3) $$B_{i}={\left[\begin{array}{ccc} R_{B}\cdot \cos\;\theta_{bi} & R_{B}\cdot \sin\;\theta_{bi} & 0 \end{array}\right]}^{T}$$

By coordinate conversion, Vector Ai is converted to in the coordinate system defined by *O-XYZ*. *L*_i_, the position vector of the *i*th leg, can be calculated as:

(4) $$L_{i}=P+RA_{i}-B_{i}$$

After differentiating Equation (4) with respect to time, velocity vectors of point Ai are:

(5) $$U_{Ai}={\dot{L}}_{i}=v+\omega \times r_{i}$$

where ***v*** and ***ω*** are the velocity and angular velocity of the moving platform respectively, and ***r**_i_* is the radius vector from point ***P*** to point ***A**_i_* after rotation of the moving platform, which can be expressed as ***r**_i_* = ***RA**_i_*.

Velocity vectors of point ***A**_i_*, which is the vectorial sum of the axial velocity and the tangential speed along the elastic leg can also be calculated as:

(6) $$U_{Ai}=l_{i}\cdot \left(v+\omega \times r_{i}\right)\cdot l_{i}+\omega_{l}\times L_{i}$$

where ***l**_i_* is the unit vector of the leg, which is expressed as $l_{i}=L_{i}/L_{i}$, and $\omega_{l}$ is the angular velocity of the leg.

**Figure 2:**
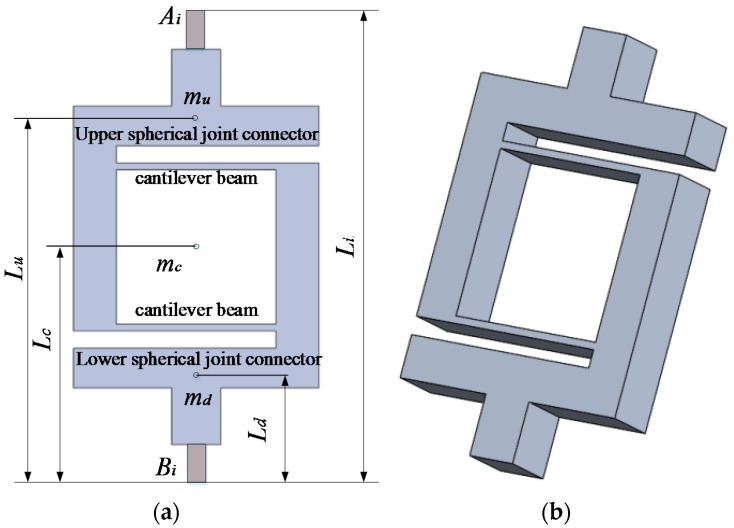
Structure of the elastic leg. (**a**) Front side of the elastic leg; (**b**) 3D model of the elastic leg.

As shown in Figure 2, the DCB elastic leg consists of three parts: an upper spherical joint connector, a double cantilever beam and a lower spherical joint connector. The mass centroids of the upper spherical joint connector, double cantilever beam and lower spherical joint connector are *m_u_*, *m_c_* and *m_d_* respectively. *L_u_*, *L_c_* and *L_d_* denote the distance between mass centroids and the connection points *B_i_* respectively.

The partial angular velocities on each point of the leg are identical. Substituting Equation (5) into Equation (6), the angular velocity of the leg is obtained by taking the cross product with ***l**_i_* on both sides of the equation:

(7) $$\omega_{l}=\left[l_{i}\times v+l_{i}\times \left(\omega \times r_{i}\right)\right]/L_{i}$$

The velocity of the mass center is the vectorial sum of the axial velocity and the tangential speed along the elastic leg on the mass centroid. The axial velocity on the mass centroid of the double flexible cantilevers is half that of the whole leg, and that on the mass centroid of the lower spherical joint connector is zero. The velocity of the mass center along the elastic leg can be calculated using:

(8) $$\{\begin{array}{l} U_{u}=l_{i}\cdot \left(v+\omega \times r_{i}\right)\cdot l_{i}+\omega_{l}\times L_{u}\\ U_{c}=\frac{l_{i}\cdot \left(v+\omega \times r_{i}\right)\cdot l_{i}}{2}+\omega_{l}\times L_{c}\\ U_{d}=\omega_{l}\times L_{d} \end{array}$$

The Lagrange equation of the accelerometer can be written as:

(9) $$\frac{d}{dt}\left(\frac{\partial E_{T}}{\partial {\dot{q}}_{j}}\right)-\frac{\partial E_{T}}{\partial q_{j}}=Q_{j}$$

where *E_T_* is the kinetic energy of the system, and *q*_j_ and ${\dot{q}}_{j}$ are the *j*th generalized coordinates and generalized speed, respectively. *Q_j_* is the generalized force. The moving parts of the system are the six elastic legs and the moving platform, and each elastic leg consists of three moving parts. The kinetic energy of the system is a summation of the translational kinetic energy and rotational kinetic energy of the 19 moving parts, which can be expressed as:

(10) $$E_{T}=\sum_{i=1}^{19}\frac{1}{2}\left({U_{i}}^{T}m_{i}U_{i}+{\omega_{li}}^{T}I_{i}\omega_{li}\right)$$

where *m_i_* and ***U**_i_* are the mass and velocity vector of the *i*th part, and ***I**_i_* and ***ω**_li_* are the moment of inertia and angular velocity vector of the *i*th part, respectively. According to the differential transformation, $\frac{d}{dt}\left(\frac{\partial E_{T}}{\partial {\dot{q}}_{j}}\right)$, $\frac{\partial E_{T}}{\partial q_{j}}$ and *Q_j_* can be calculated as:

(11) $$\{\begin{array}{l} \frac{\partial E_{T}}{\partial {\dot{q}}_{j}}=\sum_{i=1}^{19}\frac{1}{2}\left(\frac{\partial {U_{i}}^{T}}{\partial {\dot{q}}_{j}}m_{i}U_{i}+{U_{i}}^{T}m_{i}\frac{\partial U_{i}}{\partial {\dot{q}}_{j}}+\frac{\partial {\omega_{li}}^{T}}{\partial {\dot{q}}_{j}}I_{i}\omega_{li}+{\omega_{li}}^{T}I_{i}\frac{\partial \omega_{li}}{\partial {\dot{q}}_{j}}\right)\\ \frac{d}{dt}\left(\frac{\partial E_{T}}{\partial {\dot{q}}_{j}}\right)=\sum_{i=1}^{19}\left(\frac{\partial {U_{i}}^{T}}{\partial {\dot{q}}_{j}}m_{i}\frac{\partial U_{i}}{\partial {\dot{q}}_{j}}+\frac{\partial {\omega_{li}}^{T}}{\partial {\dot{q}}_{j}}I_{i}\frac{\partial \omega_{li}}{\partial {\dot{q}}_{j}}\right)\cdot {\ddot{q}}_{j}\\ \frac{\partial E_{T}}{\partial q_{j}}=\sum_{i=1}^{19}\frac{1}{2}\left(\frac{\partial {U_{i}}^{T}}{\partial q_{j}}m_{i}U_{i}+{U_{i}}^{T}m_{i}\frac{\partial U_{i}}{\partial q_{j}}+\frac{\partial {\omega_{li}}^{T}}{\partial q_{j}}I_{i}\omega_{li}+{\omega_{li}}^{T}I_{i}\frac{\partial \omega_{li}}{\partial q_{j}}\right)\\ Q_{j}=\sum_{i=1}^{19}\frac{\partial {U_{i}}^{T}}{\partial q_{j}}\cdot m_{i}g+\sum_{l=1}^{6}\frac{\partial {U_{l}}^{T}}{\partial q_{j}}F_{l} \end{array}$$

where ***g*** is the gravity vector and ***F**_l_* is the force of the *l*th elastic leg. Considering all six generalized coordinates, Lagrange equations of all the generalized coordinates are translated into differential equations for the motion:

(12) $$Μ\ddot{q}+C\dot{q}+Kq=F$$

where ***M*** is the mass matrix, ***C*** is the damping matrix, ***K*** is the stiffness matrix and ***F*** is the force vector acting on the system. Because of the minimal speed on the elastic leg, the damping matrix that is related to ***U**_i_* and ***ω**_li_* can be ignored in the motion differential equation, which may then be simplified to:

(13) $$M\ddot{q}+Kq=0$$

### 3.2. Mass Matrix of the System

Referring to Equation (11), the mass matrix can be calculated as:

(14) $$M=\sum_{i=1}^{19}(U_{i,\dot{q}}^{T}m_{i}U_{i,\dot{q}}+\omega_{li,\dot{q}}^{T}I_{i}\omega_{li,\dot{q}})$$

where $U_{i,\dot{q}}$ is the velocity Jacobian matrix of the *i*th part, derived from the velocity vector on the mass centroid with respect to the generalized speed, and $\omega_{li,\dot{q}}$ is the angular velocity matrix of the *i*th part, derived from the angular velocity vector of each part with respect to the generalized speed.

The mass of the moving platform can be calculated using the sphere quality function $m=\frac{4}{3}\pi r^{3}\rho$, where m is the quality of the sphere, *r* is the radius of the moving platform and *ρ* is the density of the material. The moment of inertia along the *x*-, *y*-, and *z*-axes is given by $I_{x}=I_{y}=I_{z}=\frac{2}{5}mr^{2}$.

The mass and moment of inertia of the elastic leg is obtained through the Solidworks environment. Based on the parallel axis theorem, the moment of inertia of the parts with respect to the base coordinate can be expressed as:

(15) $$I_{i}=T_{i}I_{io}T_{i}^{T}+m_{i}diag(d_{x}^{2},d_{y}^{2},d_{z}^{2})$$

where ***I**_io_* is the moment of inertia with respect to the mass center; *d_x_*, *d_y_* and *d_z_* are the distances between the mass center of each part and point *O* along the *X*, *Y*, *Z* axis, respectively; ***T**_i_* is the rotation matrix which rotates the local coordinate system on the lower spherical joint connector to the base coordinate system; and *m_i_* is the mass of the *i*th part.

The velocity Jacobian matrix and angular velocity matrix of the moving platform are formed by differentiating Equation (1) with respect to q and $\dot{q}$, respectively:

(16) $$U_{m,\dot{q}}=\left[\begin{array}{cc} I_{3} & 0_{3\times 3} \end{array}\right]$$

(17) $$\omega_{m,\dot{q}}=\left[\begin{array}{cc} 0_{3\times 3} & I_{3\times 3} \end{array}\right]$$

By differentiating Equation (7) with respect to $\dot{q}$, the angular velocity matrix of the elastic leg is obtained:

(18) $$\omega_{l,\dot{q}}=\left[\begin{array}{cc} {\hat{l}}_{i} & -{\hat{l}}_{i}{\hat{r}}_{i} \end{array}\right]/L_{i}$$

where the matrix marked by “^” is an anti-symmetric matrix of the vector, such as $\hat{u}=\left[\begin{array}{ccc} 0 & -u_{z} & u_{y}\\ u_{z} & 0 & -u_{x}\\ u_{y} & u_{x} & 0 \end{array}\right]$, where u×c can be expressed as $\hat{u}\cdot c$.

Differentiating Equation (8) with respect to $\dot{q}$, the velocity Jacobian matrix of the mass center along the elastic leg is obtained:

(19) $$\{\begin{array}{l} U_{u,\dot{q}}=\left[\begin{array}{cc} l_{i}\cdot {l_{i}}^{T}+\frac{L_{u}}{L_{i}}{\hat{l}}_{i}{\hat{l}}_{i} & -l_{i}\cdot {l_{i}}^{T}\cdot {\hat{r}}_{i}+\frac{L_{u}}{L_{i}}{\hat{l}}_{i}\hat{l}{\hat{r}}_{i} \end{array}\right]\\ U_{c,\dot{q}}=\left[\begin{array}{cc} \frac{l_{i}\cdot {l_{i}}^{T}}{2}+\frac{L_{c}}{L_{i}}{\hat{l}}_{i}{\hat{l}}_{i} & \frac{-l_{i}\cdot {l_{i}}^{T}\cdot {\hat{r}}_{i}}{2}+\frac{L_{c}}{L_{i}}{\hat{l}}_{i}\hat{l}{\hat{r}}_{i} \end{array}\right]\\ U_{d,\dot{q}}=\left[\begin{array}{cc} \frac{L_{d}}{L_{i}}{\hat{l}}_{i}{\hat{l}}_{i} & \frac{L_{d}}{L_{i}}{\hat{l}}_{i}{\hat{l}}_{i}{\hat{r}}_{i} \end{array}\right] \end{array}$$

### 3.3. Stiffness Matrix of the System

Referring to Equations (2) and (3), the unit line vectors of the elastic legs can be calculated as

(20) $$l_{i}=\left[\begin{array}{c} s_{i}\\ s_{ri} \end{array}\right]$$

where $s_{i}=\left(A_{i}-B_{i}\right)/\left|A_{i}-B_{i}\right|$, $s_{ri}=r\times s=\left(A_{i}\times \left(A_{i}-B_{i}\right)\right)/\left|A_{i}-B_{i}\right|=\left(B_{i}\times A_{i}\right)/\left|A_{i}-B_{i}\right|$.

For the six-axis accelerometer system, the force Jacobian matrix can be obtained:

(21) $$J={\left[\begin{array}{llllll} l_{1} & l_{2} & l_{3} & l_{4} & l_{5} & l_{6} \end{array}\right]}^{T}$$

Based on the CCT, the stiffness matrix of the system is expressed as:

(22) $$K=J^{T}K_{l}J$$

where ***K**_l_* is the diagonal stiffness matrix of the elastic legs, which can be written as:

(23) $$K_{l}=diag\left(k_{1},k_{2},k_{3},k_{4},k_{5},k_{6}\right)$$

According to [2], the elastic leg force matrix can be calculated as:

(24) $$F_{l}=K_{l}Jq={\left(J^{T}\right)}^{-1}\left(-M\ddot{q}\right)$$

## 4. Analysis of the DCB Elastic Element

### 4.1. Structure of the Elastic Legs

DCB structure is an elastic-sensitive element. Compared with the single cantilever beam, DCB structure performs better elasticity and stability characteristics, and the strain value is also enhanced. In this accelerometer, the DCB structure is applied as the elastic element. With a high strain of the DCB, the sensitivity of the system and the accuracy of the sensor are improved.

**Figure 3:**
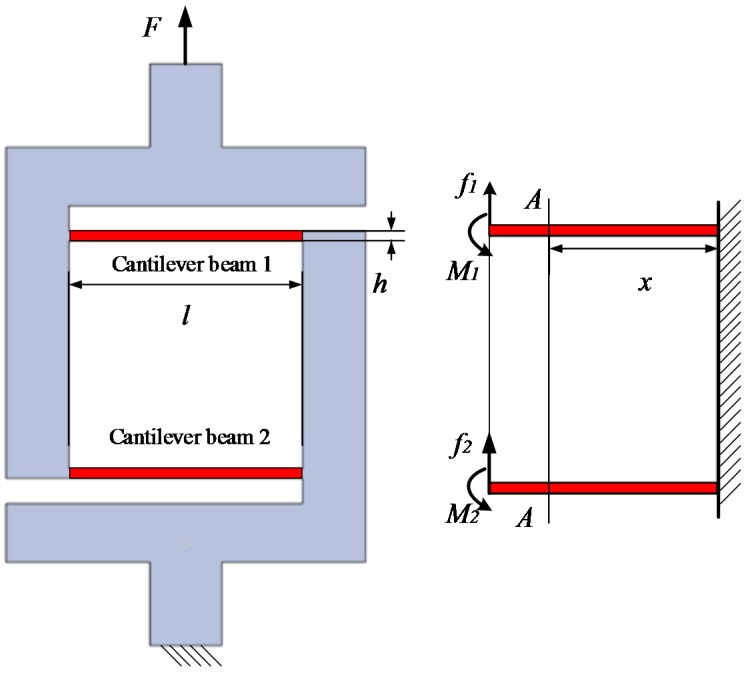
Force analysis of the double cantilever beam (DCB) elastic leg.

**Figure 4:**
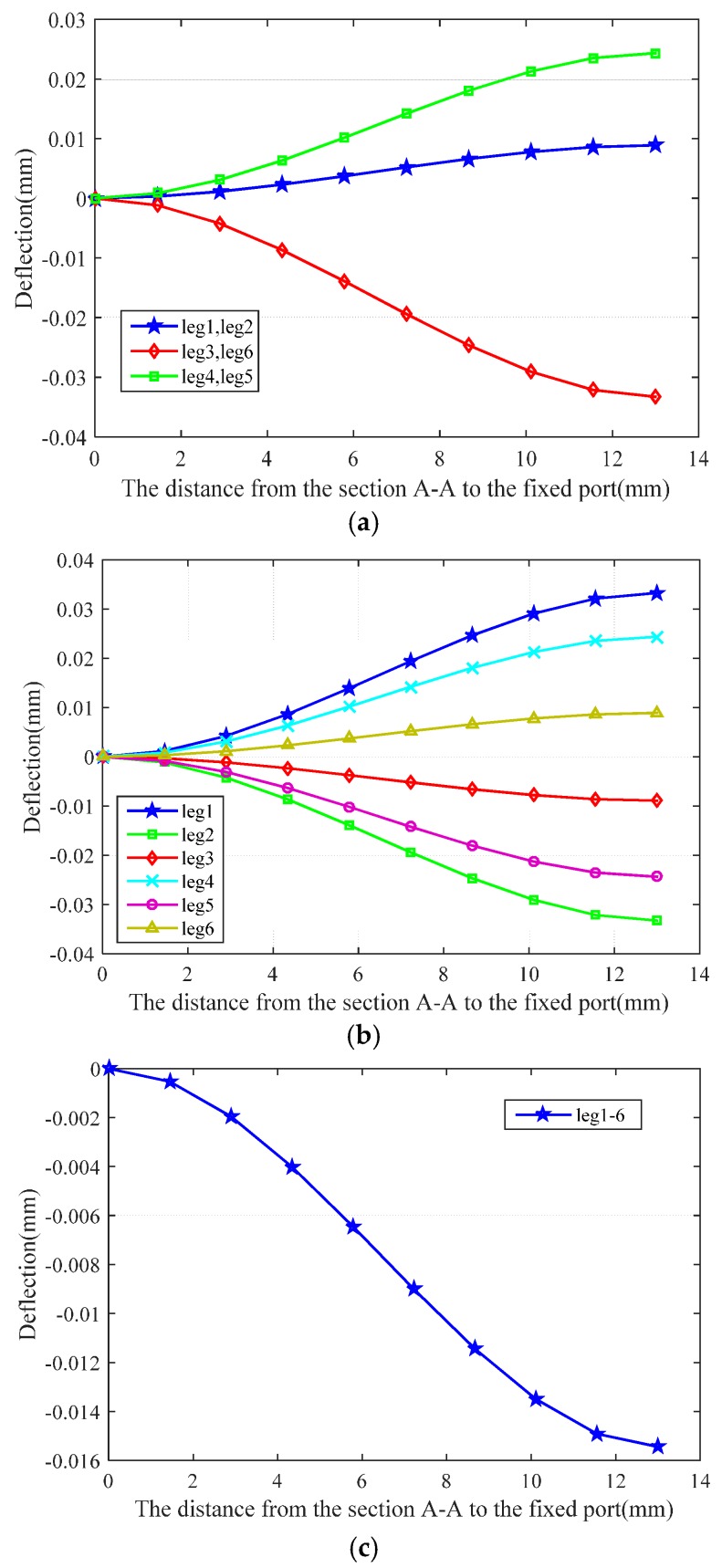
Deformation of the six beams: (**a**) deformation under a load ***g**_x_* on the moving platform; (**b**) deformation under a load ***g**_y_* on the moving platform; and (**c**) deformation under a load ***g**_z_* on the moving platform.

Figure 3 shows the force analysis of the DCB elastic leg, *F* is assumed to be a lift force on the top of the leg, and the end of the leg is fixed. The upper and lower spherical joint connectors are assumed to be rigid. The equivalent effect on the first cantilever beam is a combination of the force *f*_1_ and moment *M*_1_, and that on the second beam is *f*_2_ and *M*_2_. According to the principle of balanced forces and moments, the force equation of the DCB leg can be calculated as:

(25) $$\{\begin{array}{c} f_{1}=f_{2}=\frac{F}{2}\\ M_{1}=M_{2}=\frac{Fl}{4} \end{array}$$

where *l* is length of the cantilever beam. In Figure 4, A-A is an arbitrary cross section of the cantilever beam. Based on the differential equation of the deflection, the deflection of the section A-A is obtained:

(26) $$\{\begin{array}{c} w_{f}=\frac{Fl^{2}\left(3l-x\right)}{12EI}\\ w_{M}=\frac{M_{1}x^{2}}{2EI}=\frac{Flx^{2}}{8EI} \end{array}$$

where *I* is the moment of inertia of the cantilever beam, *w_f_* is the deformation of the cross section A-A caused by the force *F*, *w_M_* is the deformation caused by the moment *M*_1_ and *x* is the distance between the section A-A and the fixed port of the cantilever beam. Based on the moment of inertia of a rectangular section, the moment of inertia *I* can be expressed as:

(27) $$I=\frac{b\cdot h^{3}}{12}$$

where *h* is the width and *b* denotes thickness. Force analysis proves that force *F* and moment *M* have an opposite effect on the deflection. Total deflection is expressed as $w=w_{f}-w_{M}$. The total deflection of section A-A is expressed as:

(28) $$w_{A-A}=\frac{F\left(3lx^{2}-2x^{3}\right)}{24EI}=\frac{F\left(3lx^{2}-2x^{3}\right)}{2Ebh^{3}}$$

The maximum deformation of the beam is obtained when the distance between the section A-A and the fixed port equals the length of the beam. Referring to the Equations (27) and (28), the equivalent stiffness coefficient of the DCB leg can be obtained as:

(29) $$k_{l}=2\cdot \frac{F}{w}=\frac{24EI}{l^{3}}=\frac{2Ebh^{3}}{l^{3}}$$

### 4.2. Numerical Calculation

Parameters of the elastic legs are determined by the strength of the DCB structure, size of the accelerometer and the manufacturing costs. Maximum moment is at the mode point of the beam, which can be calculated as:

(30) $$M_{\text{max}}=\frac{F_{i}}{2}\cdot l-\frac{F_{i}}{4}\cdot l=\frac{F_{i}\cdot l}{4}$$

where *F_i_* is the force loading on the *i*th elastic leg, and *M_max_* is the maximum moment on the beam. Based on the formula for the normal stress of the beam, the maximum bending stress of the DCB can be calculated as

(31) $$\sigma_{\text{max}}=\frac{M_{\text{max}}\cdot h}{2I}$$

where σ_*max*_ is the maximum bending stress of the DCB. After analyzing the isotropic of the accelerometer and considering the manufacturing costs, the improved parameters of the accelerometer and the elastic legs and angle parameters of the accelerometer are obtained as shown in Table 1 and Table 2 [33].

Based on the stress requirements and the cost of component processing, the elastic element material is an aluminum alloy with elastic modulus *E* = 6.9E10 Pa. Substituting the parameters in Table 1 and Table 2 into Equation (21), the Jacobian matrix of the accelerometer can be calculated as:

(32) $$J=\left[\begin{array}{cccccc} -0.2882 & -0.2882 & -0.4550 & 0.7432 & 0.7432 & -0.4550\\ 0.6918 & -0.6918 & -0.5955 & 0.0963 & 0.0963 & 0.5955\\ -0.6621 & -0.6621 & -0.6621 & -0.6621 & -0.6621 & -0.6621\\ 0.0043 & -0.0043 & -0.0160 & -0.0117 & 0.0117 & 0.0160\\ 0.0160 & 0.0160 & -0.0043 & -0.0117 & -0.0117 & -0.0043\\ 0.0148 & -0.0148 & 0.0148 & -0.0148 & 0.0148 & -0.0148 \end{array}\right]$$

Substituting the parameters in Table 1 and Table 2 into Equation (20), the mass matrix can be calculated as:

(33) $$M=\left[\begin{array}{cccccc} 0.4425 & 0 & -0.0001 & 0 & 0 & 0\\ 0 & 0.4404 & 0 & 0 & 0 & 0\\ -0.0001 & 0 & 0.4459 & 0 & 0 & 0\\ 0 & 0 & 0 & 0.0001 & 0 & 0\\ 0 & 0 & 0 & 0 & 0.0001 & 0\\ 0 & 0 & 0 & 0 & 0 & 0.0001 \end{array}\right]$$

Substituting the parameters in Table 1 and Table 2 into Equation (22), the stiffness matrix can be calculated as:

(34) $$K=10^{5}\cdot \left[\begin{array}{cccccc} 1.1429 & 0 & 0 & 0 & -0.0154 & 0\\ 0 & 1.1429 & 0 & 0.0154 & 0 & 0\\ 0 & 0 & 1.7844 & 0 & 0 & 0\\ 0 & 0.0154 & 0 & 0.0006 & 0 & 0\\ -0.0154 & 0 & 0 & 0 & 0.0006 & 0\\ 0 & 0 & 0 & 0 & 0 & 0.0009 \end{array}\right]$$

To analyze the sensitivity of the accelerometer, acceleration components $g_{x}={\left[\begin{array}{cccccc} 9.8 & 0 & 0 & 0 & 0 & 0 \end{array}\right]}^{T}$, $g_{y}={\left[\begin{array}{cccccc} 0 & 9.8 & 0 & 0 & 0 & 0 \end{array}\right]}^{T}$ and $g_{z}={\left[\begin{array}{cccccc} 0 & 0 & 9.8 & 0 & 0 & 0 \end{array}\right]}^{T}$ are loaded onto the moving platform for deformation analysis. Substituting Equations (32)–(34) into Equation (24), the forces of the legs are calculated as:

(35) $$\{\begin{array}{c} F_{x}={\left[\begin{array}{cccccc} 0.6048 & 0.6048 & -2.2572 & 1.6524 & 1.6524 & -2.2572 \end{array}\right]}^{T}\\ F_{y}={\left[\begin{array}{cccccc} 2.2572 & -2.2572 & -0.6048 & 1.6524 & -1.6524 & 0.6048 \end{array}\right]}^{T}\\ F_{z}={\left[\begin{array}{cccccc} -1.0475 & -1.0475 & -1.0475 & -1.0475 & -1.0475 & -1.0475 \end{array}\right]}^{T} \end{array}$$

where *F_x_* is the force matrix of the legs when acceleration ***g**_x_* is loaded onto the moving platform, *F_y_* is the force matrix of the legs when acceleration ***g**_y_* is loaded on the moving platform, and *F_z_* is the force matrix of the legs when acceleration ***g**_z_* is loaded on the moving platform. A positive number indicates a tensional force, and a negative number indicates a compressional force.

Referring to Equation (28), the distance between the section A-A and the fixed port is set as the variable, and the deformation curves of the six beams are shown in Figure 4.

To validate the theoretical model, a finite element method was applied to the model and strain analysis of the system.

**Figure 5:**
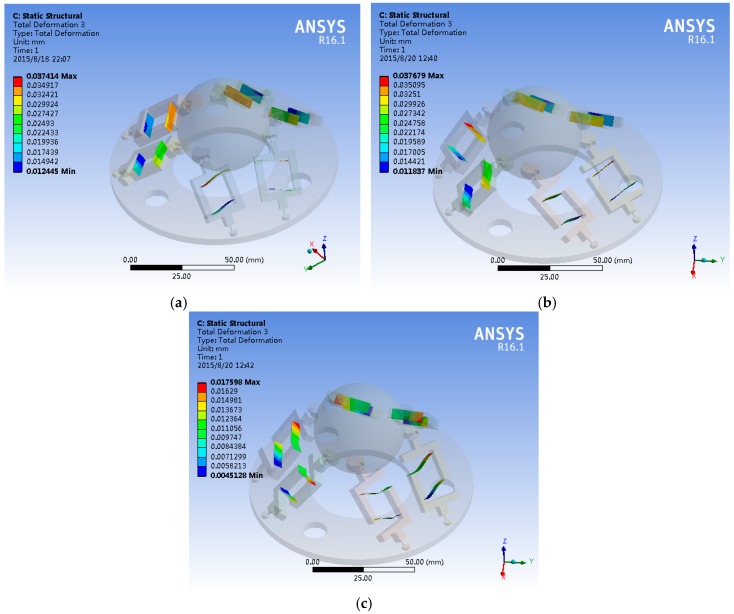
Deformation of the six beams in ANSYS Workbench: (**a**) deformation under a load ***g**_x_* on the moving platform in ANSYS Workbench; (**b**) deformation under a load ***g**_y_* on the moving platform in ANSYS Workbench; and (**c**) deformation under a load ***g**_z_* on the moving platform in ANSYS Workbench.

As shown in Figure 5, The finite element model was established in the ANSYS Workbench environment. The accelerometer was meshed using hexahedral elements, and the material of the moving platform was a copper alloy. The elastic modulus was 1.17E11 Pa, Poisson’s ratio was 0.31, and the density was 8900 kg/m^3^. The material of the elastic element was an aluminum alloy, with elastic modulus of 6.9E10 Pa, Poisson’s ratio of 0.3 and density of 2700 kg/m^3^. The base is fixed before running the model.

Accelerations ***g**_x_*, ***g**_y_* and ***g**_z_* are loaded onto the moving platform in the strain analysis. Comparing the theoretical deflection with that of the simulations, an overall trend is apparent. The maximum theoretical deflections are 3.33 × 10^−2^ mm along the *x*-axis, 3.33 × 10^−2^ mm along the *y*-axis and 1.54 × 10^−2^ mm along the *z*-axis. The maximum simulated deflections are 3.74 × 10^−2^ mm along the *x*-axis, 3.77 × 10^−2^ mm along the *y*-axis and 1.76 × 10^−2^ mm along the *z*-axis. The coincidence rate reaches 89.0% along the *x*-axis, 88.3% along the *y*-axis and 87.5% along the *z*-axis.

## 5. Theoretical Analysis of the Elastic Leg Strain

**Figure 6:**
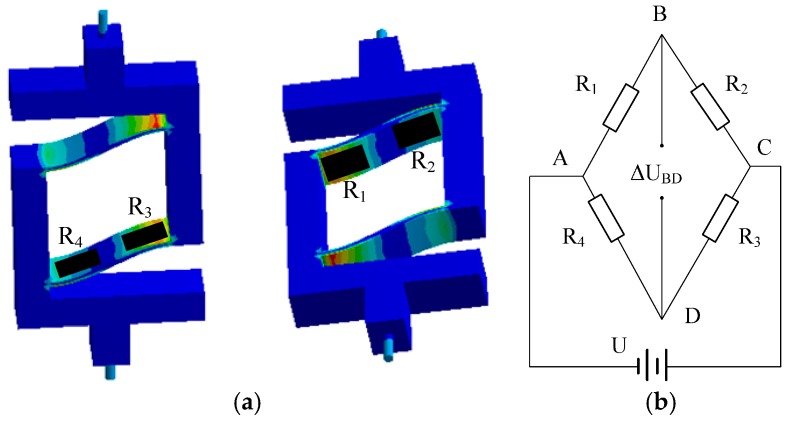
The location of the strain gauges and the bridge arrangement for one element: (**a**) the location of the strain gauges; and (**b**) bridge arrangement.

There are four sensing elements positioned at predetermined locations on the elastic leg. Figure 6 depicts the location of the strain gauges; the Wheatstone bridge arrangement for a single element is also shown. Each bridge is excited by a stable 5-V power supply. The resistances of the strain gauges are equal initially and can be described as:

(36) $$R_{1}=R_{2}=R_{3}=R_{4}=R$$

When the accelerometer is in a working state, the resistances of the strain gauges change by an amount given by Δ*R*_1_, Δ*R*_2_, Δ*R*_3_, and Δ*R*_4_. In the case where Δ*R _i_* << *R*(*i* = 1,2,3,4), the output voltage can be simplified to:

(37) $$\Delta U_{BD}=\frac{U}{4}(\frac{\Delta R_{1}}{R}-\frac{\Delta R_{2}}{R}+\frac{\Delta R_{3}}{R}-\frac{\Delta R_{4}}{R})=\frac{U}{4}K(\varepsilon_{1}-\varepsilon_{2}+\varepsilon_{3}-\varepsilon_{4})$$

where K is the sensitivity coefficient of the strain gauge and *ε_i_*(*i* = 1,2,3,4)is the strain value of each strain gauge. $\varepsilon_{1}$ and $\varepsilon_{3}$ are opposite in sign to $\varepsilon_{2}$ and $\varepsilon_{4}$, and *U* is the power supplied to the system. By making use of the integral, strain $\varepsilon_{1}$ and $\varepsilon_{3}$ are calculated using:

(38) $$\varepsilon_{1}=\frac{2\int_{0}^{\frac{l}{2}}\varepsilon_{x}dx}{l},\varepsilon_{2}=\frac{2\int_{\frac{l}{2}}^{l}\varepsilon_{x}dx}{l},\varepsilon_{3}=-\frac{2\int_{\frac{l}{2}}^{l}\varepsilon_{x}dx}{l},\varepsilon_{4}=-\frac{2\int_{0}^{\frac{l}{2}}\varepsilon_{x}dx}{l}$$

where $\varepsilon_{x}=\frac{hM_{x}}{2IE}$ is the strain of the beam on section A-A and *M_x_* is the bending moment on section A-A, which can be calculated as $M_{x}=\frac{F\left(l-2x\right)}{4}$. The strain of the beam can be expressed as:

(39) $$\{\begin{array}{c} \varepsilon_{1}=\varepsilon_{3}=\frac{3Fl}{4Ebh^{2}}\\ \varepsilon_{2}=\varepsilon_{4}=-\frac{3Fl}{4Ebh^{2}} \end{array}$$

Referring to Equation (39), the strain of one strain gauge in the elastic element is 8.22 × 10^−2^ mm with the accelerometer ***a**_z_* = 1 g. Compared with the reference 20 (With acceleration ***a**_z_* = 1 g, maximal positive strain of the accelerometer is 6.903 × 10^−5^ mm), DCB structure shows better strain performance. Measurement of the strain gauge shows that the sensitivity coefficient *K* is 4. The output voltage can be expressed as:

(40) $$\Delta U_{BD}=\frac{3UKFl}{4Ebh^{2}}$$

Sensitivities of the accelerometer means the output voltage of each elastic leg in the unit acceleration. Referring to Equations (24) and (40), the ratio of the output voltage to the acceleration can be calculated. Theoretical sensitivities of the accelerometer along the *x*-axis, *y*-axis and *z*-axis were obtained as:

(41) $$\{\begin{array}{c} \Delta {U_{x}}^{\prime }=\left[\begin{array}{cccccc} 0.0969 & 0.0969 & 0.3616 & 0.2647 & 0.2647 & 0.3616 \end{array}\right]\\ \Delta {U_{y}}^{\prime }=\left[\begin{array}{cccccc} 0.3616 & 0.3616 & 0.0969 & 0.2647 & 0.2647 & 0.0969 \end{array}\right]\\ \Delta {U_{z}}^{\prime }=\left[\begin{array}{cccccc} 0.1678 & 0.1678 & 0.1678 & 0.1678 & 0.1678 & 0.1678 \end{array}\right] \end{array}$$

where Δ*U*_x_’, Δ*U*_y_’ and Δ*U*_z_’ are the theoretical sensitivity matrixes of the elastic legs along the *x*-, *y*- and *z*-axes, respectively, in units of mV/(m/s^2^).

## 6. Sensitivity Experiments

**Figure 7:**
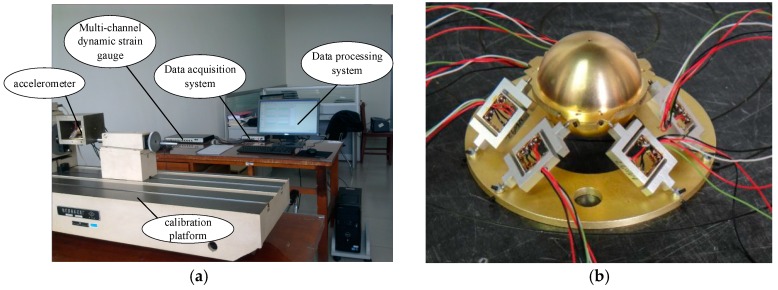
Calibration test for linear sensitivity of the accelerometer: (**a**) the calibration system for the six-axis accelerometer; and (**b**) prototype of the elastic element of the six-axis accelerometer.

In order to verify the sensitivity of the accelerometer, a calibration test on the linear sensitivity of the accelerometer was conducted (certificate number: LSzb2011-0416). As shown in Figure 7, the test system consisted of five parts: an accelerometer, multi-channel dynamic strain gauge, data acquisition system, data processing system and calibration platform. Strain gauges are located on the accelerometer and Wheatstone bridge circuit is applied in the system. The multi-channel dynamic strain gauge can achieve calibration of 12 channels simultaneously and transfer the analog signals into digital signals to a computer. The data acquisition system was connected to a microcomputer data processing system through a USB interface. The frequency of vibration standard device APS 129 ELECTRO-SEIS is applied to the calibration platform.

A calibration test on the linear sensitivity of the accelerometer was conducted. As shown in Figure 7, the test system consisted of five parts: an accelerometer, multi-channel dynamic strain gauge, data acquisition system, data processing system and calibration platform. The multi-channel dynamic strain gauge can achieve calibration of 12 channels simultaneously and transfer this information to a computer. The data acquisition system was connected to a microcomputer data processing system through a USB interface.

The system requires preheating for 30 min during the process of demarcation and calibration. The acceleration load ***g**_z_* was divided into 10 equal points along the *z*-axis. Loading was sinusoidal with a reference frequency of 16 Hz. Output voltages of the elastic legs, magnified 1000-fold, are shown in Table 3. The sensitivity shows a strong linear relationship, which can be calculated as:

Acceleration test loads along the *x* and *y*-axis were 1 m/s^2^, divided into 10 equal points. Output voltages of the elastic legs, magnified 1000-fold, are shown in Table 4 and Table 5.

The result of the output voltage shows a strong linear relationship. Based on the arithmetic mean summation method, the sensitivity of the accelerometer can be calculated as:

(42) $$\{\begin{array}{l} \Delta U_{z}=\frac{1}{10}\sum_{i=1}^{10}\frac{U_{zi}}{a_{zi}}=\left[\begin{array}{cccccc} 0.138 & 0.144 & 0.116 & 0.129 & 0.137 & 0.132 \end{array}\right]\\ \Delta U_{x}=\frac{1}{10}\sum_{i=1}^{10}\frac{U_{xi}}{a_{xi}}=\left[\begin{array}{cccccc} 0.083 & 0.096 & 0.402 & 0.344 & 0.370 & 0.416 \end{array}\right]\\ \Delta U_{y}=\frac{1}{10}\sum_{i=1}^{10}\frac{U_{yi}}{a_{yi}}=\left[\begin{array}{cccccc} 0.473 & 0.473 & 0.173 & 0.273 & 0.344 & 0.145 \end{array}\right] \end{array}$$

where Δ***U***_z_, Δ***U***_x_, and Δ***U***_y_ are the sensitivity matrix of the elastic legs along the *z*-, *x*- and *y*-axis, in units of V/m/s^2^; ***U***_zi_, ***U***_xi_, and ***U***_yi_ are the output voltage matrix of the elastic legs in the *i*th experiment; and ***a***_zi_, ***a***_xi_, and ***a***_yi_ are the acceleration loaded on the system in the *i*th experiment, respectively.

To verify the theoretical model, theoretical result magnified 1000-fold is compared with that of the experiment. Based on the arithmetic mean summation method, theoretical sensitivity accuracies along the *x*-, *y*- and *z*-axes of 90.4%, 74.9% and 78.9% with respect to the test data are obtained.

Acceleration loading times of the experiments is set as *n* = 10. Based on the arithmetic mean summation method, the measurement precision of the accelerometer is obtained in Table 6.

As can be seen in Table 6, the maximum errors of *a_x_*, *a_y_* and *a_z_* are 2.6%, 0.6% and 1.31% respectively. Table 7 shows the measurement precision of another accelerometer with parallel structure [34]. The minimum error of *a_x_*, *a_y_* and *a_z_* are 5.058%, 6.99% and 5.984% respectively. Compared with the data in Table 7, the experiments data in Table 6 prove that accelerometer with DCB elastic element performs a great sensitive and precision characteristics.

## 7. Conclusions

A theoretical model of a six-axis accelerometer with a DCB elastic element is presented based on a parallel mechanism. FEA simulations and prototype experiments prove that the accelerometer shows high sensitivity performance. The main conclusions are:

- In this paper, a dynamics model for the accelerometer is established based on the Lagrange equation, and the mass and stiffness matrices obtained. Based on structural mechanics, characteristics of the DCB elastic element are obtained. A theoretical model of the whole accelerometer is calculated.
- Accelerations ***g**_x_*, ***g**_y_* and ***g**_z_* are loaded onto the moving platform, and the deformation of the elastic legs calculated. By comparison with the FEA simulation, the coincidence rate of the deformation reached 89.0% along the *x*-axis, 88.3% along the *y*-axis and 87.5% along the *z*-axis.
- The prototype experiments show that the six-axis accelerometer with the DCB elastic element is highly sensitive. Compared to theoretical sensitivities, the accuracy of the *x*-, *y*- and *z*-axes measurements was found to be 84.6%, 75.1% and 79.8%, respectively. Prototype experiments prove the reliability of the theoretical model. The measurement errors of linear accelerations ax, ay and az are 2.6%, 0.6% and 1.31%, respectively.
- Because of the limited equipment, only linear acceleration can be loaded in the accelerometer calibration experiment. The analysis and results of the experiments are for the three linear directions only.

## Figures and Tables

**Table 1:** Parameters of the six-axis accelerometer.

Parameter	Value (mm)
*R_A_*	25
*R_B_*	52
*r*	22.5
*H*	30
*b*	5
*h*	0.6
*l*	13

**Table 2:** Angle parameters of the six-axis accelerometer.

Sequence Number	*θ_ai_*	*θ_bi_*
*i* = 1	−π/12	−π/4
*i* = 2	π/12	π/4
*i* = 3	7π/12	5π/12
*i* = 4	3π/4	11π/12
*i* = 5	5π/4	13π/12
*i* = 6	17π/12	19π/12

**Table 3:** Output voltage of each channel on *z*-axis acceleration.

No.	Acceleration (m/s^2^)	Output Voltage (mv)					
		Leg 1	Leg 2	Leg 3	Leg 4	Leg 5	Leg 6
1	1	132.4	143.8	116	128.2	137.8	131.6
2	2	277.8	289.2	232.4	258.8	274	262.8
3	3	414	434.4	348.3	387	412.5	394.5
4	4	554	578.4	465.2	519.6	552.4	526.8
5	5	690	721	581.5	646	688	660
6	6	837	867.6	696.6	777	824.4	796.2
7	7	970.2	1012.2	814.8	906.5	964.6	928.9
8	8	1110.4	1155.2	932	1036	1098.4	1060.8
9	9	1249.2	1298.7	1046.7	1163.7	1237.5	1198.8
10	10	1389	1442	1165	1292	1376	1331

**Table 4:** Output voltage of each channel on *x*-axis acceleration.

No.	Acceleration (m/s^2^)	Output Voltage (mv)					
		Leg 1	Leg 2	Leg 3	Leg 4	Leg 5	Leg 6
1	0.1	8.41	9.43	40.03	34.51	37.29	41.92
2	0.2	17.06	18.9	80.24	69.18	74.54	83.46
3	0.3	25.68	28.62	120.72	103.11	111.87	125.01
4	0.4	34.16	38.04	160.8	137.72	148.72	166.28
5	0.5	42.1	47.8	200.8	172	185.5	207.5
6	0.6	50.04	58.26	241.08	206.46	221.7	249.06
7	0.7	56.49	69.16	281.54	240.66	258.79	290.08
8	0.8	65.68	78	321.28	275.28	294.32	331.6
9	0.9	73.35	87.93	361.62	309.42	331.38	372.78
10	1	80.4	98.6	401.3	343.5	367.4	414.3

**Table 5:** Output voltage of each channel on *y*-axis acceleration.

No.	Acceleration (m/s^2^)	Output Voltage (mv)					
		Leg 1	Leg 2	Leg 3	Leg 4	Leg 5	Leg 6
1	0.1	47.22	47.43	17.28	27.47	34.79	14.49
2	0.2	94.44	94.68	34.9	55.3	69.48	28.96
3	0.3	142.08	142.5	51.66	82.35	103.56	43.62
4	0.4	189.48	188.68	69.4	109.28	138.04	58.2
5	0.5	236.55	236.15	86.7	136.55	172.1	72.85
6	0.6	283.8	284.04	103.92	163.74	206.4	87.36
7	0.7	331.38	330.68	121.52	190.26	240.66	101.92
8	0.8	378.64	377.76	138.8	217.52	275.04	116.56
9	0.9	425.61	424.53	156.42	244.26	308.88	131.04
10	1	472.8	472.2	173.8	271.2	342.9	145.6

**Table 6:** Measurement precision of the six-axis accelerometer.

Acceleration (m/s^2^)	*a_x_* Error (%) n = 10	*a_y_* Error (%) n = 10	Acceleration (m/s^2^)	*a_z_* Error (%) n = 10
0.1	2.6	0.6	1	1.31
0.2	2.55	0.5	2	1.22
0.3	2.5	0.57	3	1.1
0.4	2.45	0.53	4	1.11
0.5	2.36	0.52	5	1.23
0.6	2.45	0.52	6	1.16
0.7	2.47	0.5	7	1.02
0.8	2.48	0.5	8	1.11
0.9	2.48	0.53	9	1.15
1	2.44	0.52	10	1.11
Max Error (%)	2.6	0.6		1.31

**Table 7:** Measurement precision of another parallel type six-axis accelerometer.

Acceleration (m/s^2^)	*a_x_* Error (%)	*a_y_* Error (%)	*a_z_* Error (%)
0.15 g	5.058	7.428	\
0.30 g	\	\	6.017
1.38 g	6.899	\	7.470
2.75 g	\	6.99	\
3.82 g	\	7.108	5.984
7.64 g	7.479	\	\
Min Error (%)	5.058	6.99	5.984
